# Evaluation of corneal nerves and dendritic cells by in vivo confocal microscopy after Descemet’s membrane keratoplasty for bullous keratopathy

**DOI:** 10.1038/s41598-022-10939-w

**Published:** 2022-04-28

**Authors:** Toshiki Shimizu, Takahiko Hayashi, Atsuyuki Ishida, Akira Kobayashi, Takefumi Yamaguchi, Nobuhisa Mizuki, Kenji Yuda, Satoru Yamagami

**Affiliations:** 1 Division of Ophthalmology, Department of Visual Sciences, Nihon University School of Medicine, Ohyaguchikami-machi 30-1, Itabashi-ku, Tokyo, 173-8610 Japan; 2 Department of Ophthalmology, Yokohama City University School of Medicine, Yokohama, Kanagawa Japan; 3 Kikuna Yuda Eye Clinic, Yokohama, Kanagawa Japan; 4 Department of Ophthalmology, Graduate School of Medical Science, Kanazawa University, Ichikawa, Japan; 5 Department of Ophthalmology, Tokyo Dental College Ichikawa General Hospital, Ichikawa-shi, Chiba Japan

**Keywords:** Optical techniques, Optics and photonics, Visual system

## Abstract

This study evaluated changes in corneal nerves and the number of dendritic cells (DCs) in corneal basal epithelium following Descemet membrane endothelial keratoplasty (DMEK) surgery for bullous keratopathy (BK). Twenty-three eyes from 16 consecutive patients that underwent DMEK for BK were included. Eyes of age-matched patients that underwent pre-cataract surgery (12 eyes) were used as controls. In vivo confocal microscopy was performed pre- and postoperatively at 6, 12, and 24 months. Corneal nerve length, corneal nerve trunks, number of branches, and the number of DCs were determined. The total corneal nerve length of 1634.7 ± 1389.1 μm/mm^2^ before surgery was significantly increased in a time-dependent manner to 4485.8 ± 1403.7 μm/mm^2^, 6949.5 ± 1477.1 μm/mm^2^, and 9389.2 ± 2302.2 μm/mm^2^ at 6, 12, and 24 months after DMEK surgery, respectively. The DC density in BK cornea pre- and postoperatively at 6 months was significantly higher than in the controls, and decreased postoperatively at 12 and 24 months and was significantly lower than that at 6 months postoperatively. Thus, our results suggest that DMEK can repair and normalize the corneal environment.

## Introduction

Descemet membrane endothelial keratoplasty (DMEK) is performed as a standard procedure for Fuchs endothelial corneal dystrophy (FECD) and bullous keratopathy (BK) without severe scarring^1–6^. In the DMEK procedure, a folded sheet of the Descemet's membrane and endothelium is inserted, and its merits have been widely reported. Compared with other keratoplasties, DMEK has several advantages, such as excellent and quick visual recovery and a very low rate of allograft rejection^1,3,7^. Therefore, the number of DMEK procedures has increased in recent years, especially in Europe and the United States. Although in Japan, BK is the most common indication for corneal transplantation, the DMEK procedure is being increasingly reported and is becoming more widespread^5,8–10^. Despite the report of initial endothelial cell density (ECD) loss within a 5-year follow-up, the ECD loss could stabilize over the year, and be superior to other types of keratoplasty such as Descemet stripping automated endothelial keratoplasty (DSAEK) and PKP^11^. Despite these merits, increasing higher order aberrations, corneal nerve abnormality, and cystoid macular edema (CME) occur after DMEK surgery^4,12,13^.

Dendritic cells (DCs) are one of the three professional antigen-presenting cells that have an important role in the immune reaction and immune tolerance in peripheral tissues. In healthy human corneas, a greater number of DCs reside in the peripheral corneal epithelium than in the central area^14^. The inflammatory cytokine influenced the activation and mutated DCs^15^. The resident corneal DCs become mutated and increase in number due to conditions that cause inflammation of the cornea, such as dry eye disease and infectious keratitis^16,17^. To observe DCs in the human corneal epithelium, in vivo confocal microscopy (IVCM) is used to evaluate DCs non-invasively^18,19^.

The cornea has plenty of sensory nerves. Corneal nerves are responsible for the sensations of touch or pain in the cornea and control the amount of tear secretion with their receptors. Corneal nerves are greatly involved in the homeostasis of the corneal conjunctiva surface and maintain its internal structure despite insults from external stimuli. The length of the corneal nerve is decreased by external factors such as dry eye disease, microbial infection, and diabetes mellitus. The sub-basal nerve plexus is part of corneal nerves, which is located just under the basal epithelium, anterior to the Bowman’s layer; it is the most easily imaged structure among corneal nerve structures with IVCM^13,20,21^. Improved analysis methods using IVCM have also been reported^22^.

Bucher et al. previously reported the decrease in corneal nerve length, number, trunks after DMEK in acute phase and the recovery over time, whereas their findings were about FECD^13^. Since the underlying mechanisms are different between FECD and BK, the new study evaluating the change in the corneal nerve in BK eyes would be required. Bucher et al. also reported corneal nerve alterations after DMEK for FECD and concluded that the density and function of corneal nerves diminish after DMEK^13^; furthermore, they also demonstrated that increased density of DCs correlates with corneal nerve fiber damage in both rodents and humans^23^. However, the changes in corneal nerves in eyes with BK after DMEK surgery over the long term remain unknown. BK caused strong corneal edema. We hypothesized that the corneal nerves and dendritic cells would normalize after DMEK. Moreover, no study has ever focused on the evaluation of DCs after DMEK. Therefore, the aim of this study was to evaluate long-term changes in corneal nerves and in the distribution of DCs, before and 24 months after DMEK surgery, in eyes with BK, using IVCM.

## Results

Twenty-three eyes of 16 patients (14 women and 2 men; 74.9 ± 8.9 years, range 56–85 years) were included in this study. All patients were Asian; patient characteristics are presented in Table 1. All eyes had undergone uneventful DMEK surgery. Primary graft failure, immunological rejection, and secondary glaucoma did not occur after the surgery, but four of 23 eyes (17.3%) showed CME on OCT postoperatively. Six eyes needed rebubbling, and they showed a significant improvement of corneal clearance after that.

**Table 1:** Patient characteristics.

	Control group		DMEK group			p-value
Age (in years)	69.5 ± 8.6		74.9 ± 8.9			p = 0.12
Sex (male:female)	6: 6		2: 14			p = 0.04

		Pre-DMEK	Post-DMEK 6 months	Post-DMEK 12 months	Post-DMEK 24 months	
BCVA (logMAR)		0.83 ± 0.56	0.09 ± 0.18	0.05 ± 0.15	0.05 ± 0.13	p < 0.001, respectively*
CCT (μm)		694.3 ± 81.4	504.9 ± 45.6	508.5 ± 47.4	506.2 ± 53.7	p < 0.001, respectively*
ECD (cells/mm^2^)		2643.6 ± 128.6	1606.3 ± 526.2	1401.3 ± 530.9	1099.5 ± 463.9	p < 0.001, respectively*
Corneal sensitivity (mm)		5.2 ± 0.92	4.7 ± 1.7	4.12 ± 2.46	5.1 ± 0.7	NS

*DMEK* Descemet’s membrane endothelial keratoplasty, *BCVA* best-corrected visual acuity, *CCT* central corneal thickness, *ECD* endothelial cell density, *NS* not significant.

*Significant difference compared to pre-DMEK (p < 0.05) followed by Kruskal–Wallis rank sum test.

Values are expressed as mean ± standard deviation.

BCVA, CCT, and ECD significantly improved relative to the preoperative findings, at all measurement time points (p < 0.0001, respectively) (Table 1). The preoperative BCVA (0.82 ± 0.52 logMAR; mean ± SD) improved gradually to 0.05 ± 0.12 logMAR, 0.02 ± 0.11 logMAR, and 0.02 ± 0.11 logMAR at 6, 12, and 24 months, respectively. The postoperative CCT (504.9 ± 45.6 μm, 508.5 ± 47.4 μm, and 506.2 ± 53.7 μm at respective postoperative 6, 12, and 24 months) was significantly thinner than the preoperative CCT (694.3 ± 81.4 μm, p < 0.0001). The preoperative ECD of donor corneas was 2643.6 ± 128.6 cells/mm^2^ and postoperative ECD at 6, 12, and 24 months was 1606.3 ± 526.2 cells/mm^2^, 1401.3 ± 530.9 cells/mm^2^, and 1099.5 ± 463.9 cells/mm^2^, respectively (p < 0.0001). Although corneal sensitivity slightly decreased at 6 months after surgery, the change following surgery was not significantly difference [5.2 ± 0.93 cm, 4.1 ± 1.7 cm, 4.1 ± 2.5 cm, and 5.1 ± 0.7 cm at preoperative, 6, 12, and 24 months respectively (p > 0.05)].

**Figure 1:**
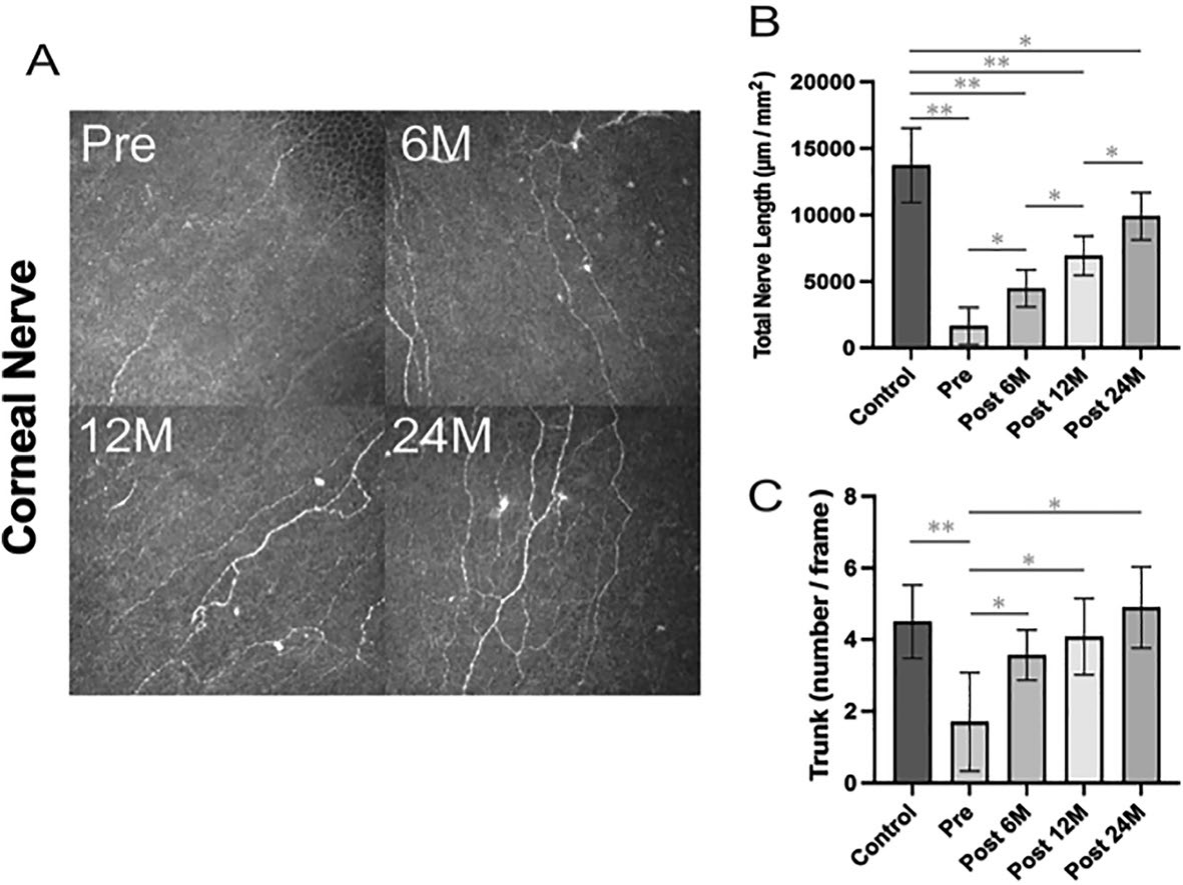
Corneal nerve length and number of nerve trunks. Total nerve length and number of nerve trunks evaluated with IVCM. Left: Representative images at evaluation points. Corneal nerve density gradually increases, and number of nerve trunks also increases after DMEK. Right up: Total nerve length in the control group is significantly more than that at other time points. Total nerve length in pre-DMEK corneas significantly elongates after DMEK for BK in a time-dependent manner. Right down: Number of nerve trunks is significantly higher post-DMEK compared with that pre-DMEK (p < 0.05). *DMEK* Descemet membrane endothelial keratoplasty, *IVCM* in vivo confocal microscopy, *Pre* pre-operative, *Post* postoperative, *M* months after surgery. *p < 0.05, **p < 0.001.

Figure 1 shows the evaluation of the corneal nerves with IVCM. As shown in the representative image of corneal nerves (Fig. 1, left), the nerves and trunks were rare in the preoperative corneal stroma, whereas the number of corneal nerves and trunks apparently increased at 6, 12, and 24 months after surgery. The total nerve length of the control group (13,725 ± 2787.8 μm/mm^2^) was significantly greater than that of the DMEK group at all measurement periods (preoperatively, p = 0.005; at 6 months postoperatively, p < 0.0001; at 12 months, p = 0.0009; at 24 months, p = 0023). The total nerve length of 1634.7 ± 1389.1 μm/mm^2^ before surgery was significantly elongated in a time-dependent manner to 4485.8 ± 1403.7 μm/mm^2^, 6949.5 ± 1477.1 μm/mm^2^, and 9389.2 ± 2302.2 μm/mm^2^ at 6, 12, and 24 months after DMEK surgery, respectively (p < 0.05) (Fig. 1, right up). Despite the gradual elongation of the total nerve length until 24 months after surgery, the total nerve length at 24 months was still significantly shorter than that in the controls (p = 0.0023). Preoperatively, the number of nerve trunks was 1.7 ± 1.4 number/frame, and this was significantly lesser than that of the controls (4.5 ± 1.0 number/frame, p = 0.0004). The number of nerve trunks gradually increased from before surgery to 24 months postoperatively (Fig. 1, right down). The number of nerve trunks significantly increased at 6, 12, and 24 months compared to those of the controls (3.6 ± 0.7, 3.7 ± 1.2, 4.9 ± 1.1 number/frame, p = 0.003, 0.017, 0.003, respectively) (Fig. 1, right down).

**Figure 2:**
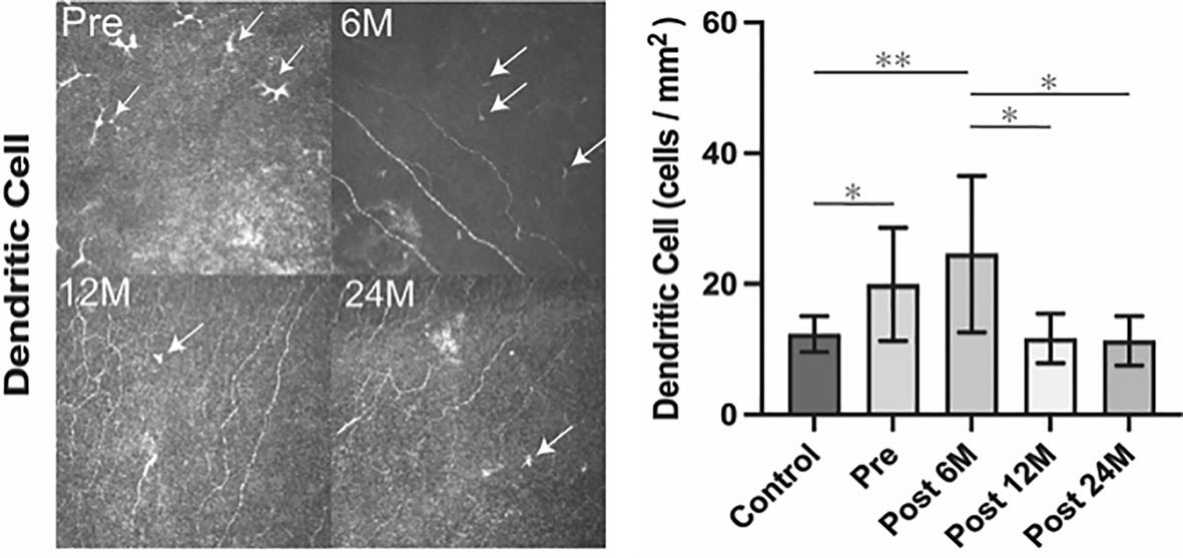
Change in dendritic cells (DCs) pre- and post-DMEK. DCs were counted with IVCM. Left: DCs (white arrows) are identified owing to high brightness and their typical dendritic morphology. Right: DCs in preoperative BK corneas are significantly more than those in controls. Number of DCs at 6 months post DMEK is significantly higher than that of controls, and at 12 and 24 months after DMEK. *BK* bullous keratopathy, *DMEK* Descemet membrane endothelial keratoplasty, *IVCM* in vivo confocal microscopy, *Pre* pre-operative, *Post* postoperative, *M* months after surgery. *p < 0.05, **p < 0.001.

Figure 2 shows the density of DCs over time, represented as images and a graph. The DC density in preoperative BK cornea (19.9 ± 8.7 cells/mm^2^) and postoperatively at 6 months (24.5 ± 11.9 cells/mm^2^) was significantly higher than that in the controls (12.3 ± 2.7 cells/mm^2^, p = 0.03, p < 0.0001, respectively). It decreased postoperatively at 12 and 24 months and was significantly lower than that at postoperative 6 months (11.7 ± 3.8 cells/mm^2^, p = 0.003; 11.3 ± 3.8 cells/mm^2^, p = 0.0006, respectively). Moreover, the density of DCs at postoperative 12 and 24 months showed similar values compared to controls.

The total nerve length and each endpoint showed a significantly weak correlation (CCT: p = 0.0013, r^2^ = 0.31. ECD: p = 0.24, r^2^ = 0.0025. Branch: p = 0.0002, r^2^ = 0.30, Trunk: p < 0.0001, r^2^ = 0.46, DCs: p = 0.0013, r^2^ = 0.22, respectively). All the endpoints showed improvement as the total nerve length was increased (Supplementary Fig. S1).

The sample size was confirmed by a power calculation. The effect size was assumed to be 2.05, based on pre-DMEK values and 6-month total nerve length density values. The calculated power for 23 eyes, which is the sample size of this study, was more than 99.9% for a two-sample t-test with a two-sided significance level of 0.05. Adding the two assumptions, the effect sizes were 3.83 and 5.58, based on 12-month and 24-month values of total nerve length density; the power curves are shown below. Under all assumptions, the calculated powers of 23 eyes were 99.9% or more. Thus, we considered this study's sample size (23 eyes) large enough to detect changes in total nerve length density after DMEK.

## Discussion

With improved techniques of corneal transplantation, there have been an increasing number of keratoplasties performed all over the world, including Japan^1,24^. Recently, endothelial keratoplasty, such as DMEK, has been recognized as the standard treatment option with excellent outcomes for endothelial dysfunction^2,5,25^. We have found significant anatomical changes in the cornea after DMEK surgery and have previously reported that significant changes in the corneal thickness or corneal volume can be attributed to a drastic change in the pathological corneal structure^26,27^. However, changes over the course of time in microstructure have not been fully elucidated in DMEK surgery for BK. In general, corneal edema may be more severe in BK than in FECD. Therefore, we tried to evaluate intra-corneal structures, especially the corneal nerves and DCs, pre- and post-DMEK surgery for BK—not for FECD and in eyes without corneal edema—using IVCM. Although the endothelial cell loss rate is higher in the high-risk eyes in this report than in other reports, the ECD value and ECD loss depend on the etiology. In fact, in PBK, one year's ECD (about 1400 mm^2^) was similar to our result. Other reports showed about 60% or more endothelial cell loss rates in DMEK for eyes with a history of glaucoma surgery or penetrating keratopathy^28,29^. The midterm results after DMEK from Ham et al. found that the ECD declines by 9% every year, although its cohort consisted of mainly FECD^30^. Asian eyes with DMEK suggested higher ECD loss^31^. The study from Asia indicated the impact of the environment of the anterior chamber in terms of inflammation and increased cytokine level^32^. Considering the etiology in our cohort (Asian patients with shallow anterior chamber or damaged iris) could also explain the slightly higher cell loss.

A major strength of this study was the precise analysis of corneal nerves and DCs using IVCM in BK. Our findings demonstrated that the number of corneal stromal nerves and nerve trunks decreased in BK and gradually increased 24 months after DMEK; the number of DCs increased transiently after DMEK surgery and then decreased gradually, suggesting that DMEK could markedly repair and normalize the corneal environment. Furthermore, there was a weak but significant correlation between DC and total, as well as between total and all endpoints without corneal sensation. To the best of our knowledge, this is the first study to have evaluated DCs as well as corneal nerves after DMEK for BK; our findings are suggestive of corneal normalization after DMEK^18^.

IVCM is useful for evaluating corneal nerves/DCs and has been reported in several studies for corneal evaluation in corneal infections and dry eye^20,33^. The corneal nerves did not normalize after PKP, even with long-term observation^34,35^. Aggarwal et al. reported a decrease in the corneal nerve density in BK compared to that in the normal cornea and in FECD^33^. In the early phase after DMEK, corneal nerve degeneration has been reported^13^. The IVCM provides only morphological data; the pathogenesis of this degeneration and reduction in the length of corneal nerves in BK remain unknown. Alomar et al. Studied changes in FECD and in patients with pseudophakic BK with corneal edema using IVCM^19^ and showed that 30% of the cases lacked corneal nerves, and the remaining 70% had decreased corneal nerve density. Al-Aqaba reported corneal nerve aberrations in BK. They suggested that the fluid between the corneal stromal cells displaces the collagen lamellae, causing corneal degeneration and denervation^18^. The decrease in corneal nerves in the present study and a similar decrease observed previously by Al-Aqaba et al. suggest that corneal edema may be one of the causes of the decrease in corneal innervation^18^.

Another important finding of this study is the dynamic change in infiltrating DCs before and after surgery. DC density was significantly higher in preoperative BK cornea and at 6 months after DMEK surgery than that in the control group, and then decreased after surgery. This may be explained by surgical invasion that causes temporary inflammation and results in an increase in the DC density, followed by a subsequent decrease due to corneal normalization post-DMEK. However, it is unclear as to why the number of DCs at 6 months was high among all measurement points despite a clear cornea with a completely attached graft. Because DCs in the corneal stroma are involved in antigen presentation and rejection^36^, the increased number of DCs at 6 months implies that some immune response still persists in the cornea and affects sensitive DCs in the microenvironment. Although allograft rejection did not occur in our cases in the observation period up to 24 months after surgery, these findings suggest that the immune response persists 6 months after DMEK, and clinicians should consider the possibility of allograft rejection.

The limitation of this study was that the underlying risk factors of nerve injury were not determined. Since various factors may be involved, examining multiple factors should be warranted. During the observational period, the total nerve length remained shorter than normal after DMEK. Therefore, it was unclear whether the total nerve length would remain short and flatten or extend until normalization with follow-up. The mechanism of the temporary increase in DCs is also unclear and should be considered in further pathological studies using postmortem tissues.

In conclusion, the length of corneal nerves gradually increased after DMEK surgery, and increased number of DCs in preoperative BK cornea persisted until 6 months postoperatively and decreased thereafter, suggesting that DMEK could repair and normalize the corneal environment.

## Methods

### Patient selection

Patients who underwent DMEK surgery at Yokohama Minami Kyosai Hospital from January 2018 to December 2020 were included in the study. The indication for DMEK was BK for all patients. Exclusion criteria were as follows: (i) patients with a history of any ocular pathology other than BK and cataract, such as severe corneal scarring, ocular trauma, severe dry eye syndrome, and contact lens use; (ii) general exclusion criteria included diabetes, any neurological disorder, or any severe systemic diseases. The exclusion criteria in (i) was adopted as the general DMEK indication criteria, and the exclusion criterion (ii) was adopted for this study to exclude the effect of neurodegeneration caused by disease. Twelve eyes of 12 patients that were healthy before the cataract surgery were evaluated as controls using IVCM. The same general exclusion criteria were applied for the control group. This prospective, nonrandomized, clinical, single-center study was approved by the Ethical Review Board of Yokohama Minami Kyosai Hospital (YKH_29_2_5) and adhered to the tenets of the Declaration of Helsinki. Written informed consent was obtained from all patients before surgery.

### Surgical procedures

All DMEK procedures were performed by the same experienced surgeon (TH), as previously described in literature^37,38^. Briefly, a pre-stripped donor tissue was punched for the estimated size (approximately 8.0-mm) with asymmetric semicircular marks on a vacuum punch (Moria Japan, Tokyo, Japan) stained with 0.1% Brilliant Blue G dye^37,38^. After removing the host’s Descemet membrane under air infusion, a DMEK graft was implanted using an IOL inserter (WJ-60M; Santen, Osaka, Japan) into the anterior chamber via a 2.8-mm-corneoscleral tunnel; it was then unfolded, and fixed with 20% SF6 gas. Peripheral iridectomy was performed at the 6 o’ clock position using a 25-gauge vitreous cutter (Stellaris PC Vitrectomy system; Bausch & Lomb, St. Louis, MO, USA).

At the end of the surgery, a subconjunctival injection of 0.4 mg of betamethasone (Rinderon; Shionogi, Osaka, Japan) was administered. Two hours after surgery, slit-lamp examination was performed. All patients were instructed to maintain the supine position for several days. Postoperative medications included a combination of 1.5% levofloxacin (Cravit; Santen), betamethasone (Sanbetason; Santen), and 2% rebamipide ophthalmic solution (Mucosta; Otsuka, Tokyo, Japan), four times daily for 3 months; the dosage was tapered thereafter. Topical tropicamide was not included in the postoperative regimen.

### Ophthalmic examinations

In all participants, the following were measured: best-corrected visual acuity (BCVA, logarithm of the minimum angle of resolution [logMAR]), intraocular pressure with non-contact tonometry, corneal sensitivity, ECD, and central corneal thickness (CCT). Corneal sensitivity was measured using an esthesiometer (Cochet-Bonnet; Luneau Technology Operations, France). Anterior segment optical coherence tomography (OCT; SS1000, Tomey Corporation, Aichi, Japan) was performed for measuring the CCT. CCT was visually checked for evaluation by the built-in software and corrected if it was off-center. OCT (RS3000, Nidek, Japan) was used to check the presence of CME. ECD was evaluated with a specular microscope at the center of the cornea (FA3509; Konan Medical Hyogo, Japan). To study the function and morphology of corneal nerves, we performed corneal sensation measurement before DMEK, and at 6, 12, and 24 months after DMEK.

### In vivo confocal microscopy

IVCM was examined in all eyes preoperatively, and 6, 12, and 24 months postoperatively as described below. Corneal nerves of all eyes were evaluated by a laser scanning confocal microscope (Heidelberg Retina Tomograph III Rostock Corneal Module; Heidelberg Engineering GmbH, Heidelberg, Germany) based on a report by Al-Aqaba^16^. Briefly, the Heidelberg Retina Tomograph III Rostock Corneal Module scanned the cornea with a field of view of 400 × 400 μm image to visualize details of corneal cells, keratocytes, and structures from the epithelium to the endothelium, including epithelial cells and the sub-basal nerve plexus. IVCM was performed under topical anesthesia with oxybuprocaine hydrochloride 0.4% (Benoxil ophthalmic solution 0.4%, Santen, Osaka). The 0.2% polyacrylic gel (Comfort gel; Bausch & Lomb, Berlin, Germany) was dropped onto the objective lens to serve as an immersion fluid.

At an area of approximately 8 mm × 8 mm at the center of the cornea, all layers of the cornea were scanned. Frames from the sub-basal layer (defined as the layer beneath basal cells of corneal epithelium) and stromal layers containing nerves were selected for analysis. One picture each from three different parts of the frames that scanned each case clearly were selected. Total nerve length is defined as total nerve length of all nerve fiber and branch within frames in mm per mm^2^. Total nerve length analysis was conducted by manually tracing all visible nerves by NeuronJ, a plug-in for ImageJ (http://www.imagescience.org/meijering/software/neuronj/). The obtained image data was measured by NeuronJ, but no image correction was performed. Standard quantitative descriptors for nerve studies were examined. Corneal nerve trunks are defined as the total number of main nerve trunks observed in one image after analyzing the images anterior and posterior to the analyzed image to confirm that these do not branch from other nerves^20^.

We determined the density of DCs (cells/mm^2^) by identifying DCs in each image at the level of the basal epithelium or at the sub-basal nerve plexus owing to their high brightness and dendritic morphology based on a report by Shetty et al.^39,40^. The images were analyzed each end points by two blinded observers, and the average of the values was used for statistical analysis.

### Data analysis

Statistical analysis was performed using JMP Pro software version 15.0.0 (SAS Institute Inc., Cary, NC, USA). We used the Mann–Whitney *U* test followed by the Kruskal–Wallis rank sum test to compare BCVA, CCT, corneal sensitivity, and ECD at all measurement points (preoperatively, and 6, 12, and 24 months after DMEK). Evaluations performed with IVCM for total nerve length, number of trunks, and number of DCs were analyzed in the control group as well as in the four aforementioned groups. The correlation between the total nerve length and each endpoint (CCT, ECD, Trunk, Branch, DCs) was analyzed. The sample size was validated with calculated power of > 99%. Statistical significance was defined as p < 0.05. All quantitative variables are expressed as the mean ± standard deviation (SD).

## Supplementary Information

Supplementary Figure S1.

## Data Availability

The datasets generated during and/or analysed during the current study are available from the corresponding author on reasonable request.
