# Supplementary material for: Evaluation of corneal nerves and dendritic cells by in vivo confocal microscopy after Descemet’s membrane keratoplasty for bullous keratopathy
Source: Sci Rep. 2022 Apr 28;12:6936. doi: 10.1038/s41598-022-10939-w (PMC9050645; doi:10.1038/s41598-022-10939-w)
Supplement: Supplementary file 1 — Supplementary Figure S1. [file 41598_2022_10939_MOESM1_ESM.pdf]

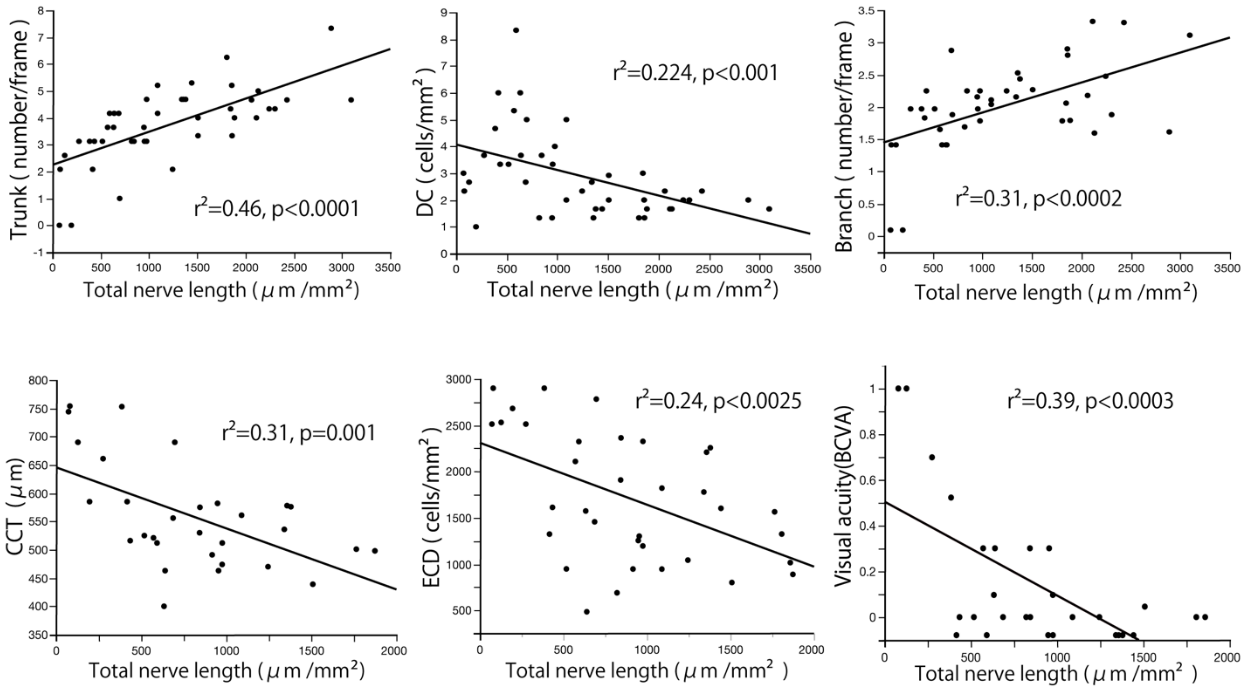

Supplementary Figure S1

Correlation between total nerve length and each endo points. Each endpoint improved in significant correlation with the increase in corneal innervation. DC: dendritic cell, CCT: central corneal thickness, ECD: endothelial cell density, BCVA: best-corrected visual acuity.
